# The development of a custom RNA-sequencing panel for the identification of predictive and diagnostic biomarkers in glioma

**DOI:** 10.1007/s11060-024-04563-z

**Published:** 2024-02-16

**Authors:** Yukina Shirai, Toshihide Ueno, Shinya Kojima, Hiroshi Ikeuchi, Rina Kitada, Takafumi Koyama, Fumiyuki Takahashi, Kazuhisa Takahashi, Koichi Ichimura, Akihiko Yoshida, Hirokazu Sugino, Hiroyuki Mano, Yoshitaka Narita, Masamichi Takahashi, Shinji Kohsaka

**Affiliations:** 1 Division of Cellular Signaling, National Cancer Center Research Institute, 5-1-1 Tsukiji, Chuo-Ku, Tokyo, 104-0045 Japan; 2 Department of Respiratory Medicine, Graduate School of Medicine, Juntendo University, 2-1-1 Hongo, Bunkyo-Ku, Tokyo, 113-8431 Japan; 3 Department of General Thoracic Surgery, Juntendo University School of Medicine, 2-1-1 Hongo, Bunkyo-Ku, Tokyo, 113-8431 Japan; 4 Department of Experimental Therapeutics, National Cancer Center Hospital, 5-1-1 Tsukiji, Chuo-Ku, Tokyo, 104-0045 Japan; 5 Department of Brain Disease Translational Research, Graduate School of Medicine, Juntendo University, 2-1-1 Hongo, Bunkyo-Ku, Tokyo, 113-8431 Japan; 6 Department of Diagnostic Pathology, National Cancer Center Hospital, 5-1-1 Tsukiji, Chuo-Ku, Tokyo, 104-0045 Japan; 7 Department of Neurosurgery and Neuro-Oncology, National Cancer Center Hospital, 5-1-1 Tsukiji, Chuo-Ku, Tokyo, 104-0045 Japan

**Keywords:** Molecular profiling, Driver mutation, RNA-seq, Glioma, Gene fusion

## Abstract

**Purpose:**

Various molecular profiles are needed to classify malignant brain tumors, including gliomas, based on the latest classification criteria of the World Health Organization, and their poor prognosis necessitates new therapeutic targets. The Todai OncoPanel 2 RNA Panel (TOP2-RNA) is a custom-target RNA-sequencing (RNA-seq) using the junction capture method to maximize the sensitivity of detecting 455 fusion gene transcripts and analyze the expression profiles of 1,390 genes. This study aimed to classify gliomas and identify their molecular targets using TOP2-RNA.

**Methods:**

A total of 124 frozen samples of malignant gliomas were subjected to TOP2-RNA for classification based on their molecular profiles and the identification of molecular targets.

**Results:**

Among 55 glioblastoma cases, gene fusions were detected in 11 cases (20%), including novel *MET* fusions. Seven tyrosine kinase genes were found to be overexpressed in 15 cases (27.3%). In contrast to isocitrate dehydrogenase (IDH) wild-type glioblastoma, IDH-mutant tumors, including astrocytomas and oligodendrogliomas, barely harbor fusion genes or gene overexpression. Of the 34 overexpressed tyrosine kinase genes, *MDM2* and *CDK4* in glioblastoma, 22 copy number amplifications (64.7%) were observed. When comparing astrocytomas and oligodendrogliomas in gene set enrichment analysis, the gene sets related to 1p36 and 19q were highly enriched in astrocytomas, suggesting that regional genomic DNA copy number alterations can be evaluated by gene expression analysis.

**Conclusions:**

TOP2-RNA is a highly sensitive assay for detecting fusion genes, exon skipping, and aberrant gene expression. Alterations in targetable driver genes were identified in more than 50% of glioblastoma. Molecular profiling by TOP2-RNA provides ample predictive, prognostic, and diagnostic biomarkers that may not be identified by conventional assays and, therefore, is expected to increase treatment options for individual patients with glioma.

**Supplementary Information:**

The online version contains supplementary material available at 10.1007/s11060-024-04563-z.

## Introduction

Gliomas, a clinically heterogeneous group of primary brain tumors, are thought to be derived from genetically or epigenetically aberrant cells with neuroglial stem/progenitor-like properties and are found in approximately 100,000 people per year worldwide [1]. They are among the most common and deadly types of primary brain tumors, accounting for approximately 28% of all brain tumors but the majority of deaths [2]. Adult gliomas are currently classified into two major groups based on the mutational status of *isocitrate dehydrogenase (IDH)1/2*, the key glioma driver gene encoding IDH [3–6]. IDH-mutant gliomas typically present as lower histologic grades with improved prognosis and a median survival of > 12 years [7]; however, later in the natural history of the disease, they often transform into higher grades with aggressive clinical behavior.

In contrast, IDH-wildtype gliomas usually present as glioblastomas (GBM), the most common and clinically aggressive World Health Organization (WHO) grade IV gliomas, with a median survival of 15–18 months despite aggressive multimodality therapy [8, 9].

Over the past decade, comprehensive molecular characterization has identified complex genetic, epigenetic, and chromosomal changes that segregate gliomas into distinct molecular subtypes, with some genetic differences affecting their response to therapy [10–12]. For example, *MGMT* promoter methylation is both prognostic and predictive of temozolomide benefits [13]. However, relatively few patients with gliomas benefit from genome-driven oncology [14, 15]. However, biomarker-driven targeted therapy has proven effective in GBM based on case stories with specific aberrations [16, 17]. This includes gene fusions that have resulted in the approval of tropomyosin receptor kinase (TRK) inhibitors for TRK fusion-positive cancers, regardless of histology [18, 19] or dabrafenib–trametinib combination for solid tumors with *BRAF* mutations [20].

As clinical sequencing is implemented in global clinical practice, the clinical utility of DNA panels that interrogate several hundred genes has been evaluated. Although RNA-sequencing (RNA-seq) technologies greatly promote the exploration of the complex and dynamic nature of cancer [21] and can provide insights into previously undetected changes occurring in a disease, the clinical utility of RNA-seq has not been comprehensively evaluated [22]. RNA-seq data have been successfully used to identify single nucleotide variant mutations [23], alternative splicing [24], fusion genes [25], and RNA editing [26].

The Todai OncoPanel (TOP) is a dual DNA–RNA panel as well as a paired tumor–normal matched test developed by our group [27]. Two hundred patients with cancer without standard treatment or those who had already undergone standard treatment underwent TOP as part of Advanced Medical Care B (UMIN000033647). The percentage of patients who received therapeutic or diagnostic recommendations was 61% (120/198 patients). One hundred and four samples (53%) harbored gene alterations that were detected using the DNA panel and had potential treatment implications. Twenty-two samples (11.1%) harbored 30 fusion transcripts or *MET* exon 14 skipping, which were detected using the RNA panel. Overall, 12 patients (6%) received recommended treatment [28]. After the trial, we revised the TOP panel to TOP2 to expand 737 gene alterations with its DNA panel and 455 fusion transcripts and 1390 gene expression with its RNA panel. For the RNA panel, probes were designed to cover fusions reported in databases, such as COSMIC (https://cancer.sanger.ac.uk/cosmic/fusion) and FusionGDB (https://ccsm.uth.edu/FusionGDB/). For gene expression, probes cover all genes targeted by the DNA panel and fusion genes, as well as genes whose protein expression is commonly evaluated by immunohistochemistry in pathological diagnosis.

As RNA-seq is a reliable method for detecting fusion genes, molecular profiling of gliomas using the TOP2 RNA Panel (TOP2-RNA) was performed in this study, and its validity and potential utility in the molecular diagnosis and stratification of patients for clinical trial enrollment were evaluated.

## Materials and methods

### Study design and patient specimens

The study cohort was comprised of 131 patients with gliomas who underwent surgical resection between 2005 and 2017 at the National Cancer Center, Japan. Seven cases with poor RNA quality isolated from the specimens were excluded. Analysis was conducted on the remaining 124 patients. Fresh frozen specimens of surgically resected tumors were obtained. This study was approved by the Ethics Committee of the National Cancer Center, Japan (No. 2013–042). All patients provided written informed consent, except for those who could not be reached because of loss to follow-up or death after registration. In these cases, the Institutional Review Board at the National Cancer Center granted permission to use existing tissue samples for research. No samples from patients who opted out of participation were used.

### RNA-seq with the TOP2 cancer gene panel for mutation calls

Total RNA was extracted from fresh frozen samples using the RNeasy Mini Kit (QIAGEN, Hilden, Germany). Then, 200 ng of RNA was converted to cDNA using the ProtoScript II First Strand cDNA Synthesis Kit and NEBNext Ultra II Non-Directional RNA Second Strand Synthesis Module (New England Biolabs, Ipswich, MA, USA) and subjected to subsequent target enrichment using TOP-RNA-V6 probes with the Twist Library Preparation EF Kit (Twist Bioscience, South San Francisco, CA, USA). Using a paired-end option, massive parallel sequencing of the isolated fragments was conducted using a NovaSeq 6000 (Illumina). Paired-end reads containing masked nucleotides with a quality score < 20 were aligned to the human reference genome (hg38) using STAR (v2.5.2a; https://github.com/alexdobin/STAR). Mutations were called using an in-house mutation caller based on the SAMtools’ mpileup results. Mutations were discarded if any of the following criteria were met: read depth < 20, variant allele frequency < 0.001, and the presence of the variant in normal human genomes in either the 1000 Genomes Project dataset (https://www.internationalgenome.org/) or our in-house database. Gene mutations were annotated using SnpEff (http://snpeff.sourceforge.net).

### Detection of fusion genes and exon skipping, and expression analysis

Gene fusion was detected using STAR-Fusion (v1.2.0; https://github.com/STAR-Fusion) and an in-house pipeline. The detection criterion was set to ≥ 10 reads. In the in-house pipeline, BWA (v0.7.12; https://bio-bwa.sourceforge.net/) was used to map hypothetical fusion gene sequences obtained from the reported fusion gene information. Reads matching ≥ 30 bp from the breakpoints were considered. This pipeline can also be used to identify exon skipping based on the same principle. For expression-level analysis, transcripts per million (TPM) of 1,390 genes from BAM files mapped to hg38 with STAR were calculated using an in-house program.

### Clustering and prognostic marker identification using expression data

All statistical analyses were performed using R software version 4.2.0 and related packages. Hierarchical clustering was performed using Ward’s method with log-transformed TPM as the input. In survival assessment, overall survival (OS) period was defined as the time from the start of treatment to the date of death from any cause or the date of the last follow-up. Cox regression was performed using the RegParallel (1.14.0) package, with log-transformed TPM converted to Z-scores as the input, and each of the seven genes was *p* < 0.001. For *ATXN3* expression, which was the most significant, survival curves were generated for the two groups, > 0 and < 0, and evaluated using the log-rank test.

### Copy-number analysis using digital droplet polymerase chain reaction (ddPCR)

DNA samples were analyzed by measuring concentrations using a Qubit 3.0 fluorometer (Thermo Fisher Scientific), and those that met the quality evaluation criteria (DNA quantity and quantity) were used. The reaction mix for the assay was prepared as follows: 20 ng of DNA per reaction, 1.1 μL of probes for individual target genes and the control reference gene, 11.0 μL of ddPCR Supermix, 0.5 μL of restriction enzyme, DNA up to 8 μL, and DNase-free water up to 22 μL. DNase-free water was used as the negative control for the assay, and human reference genomic DNA (NA18943; Coriell Institute, Camden, NJ) was used as the positive control. The reaction mixture was subjected to restriction enzyme treatment (MseI, R0525L, New England Biolabs, Ipswich, MA, USA), followed by droplet generation using an automated droplet generator (Bio-Rad Laboratories Inc., Hercules, CA, USA) and PCR using a Veriti Thermal Cycler (Thermo Fisher Scientific). After PCR, the fluorescence signal of each droplet was measured using a QX200 Droplet Reader (Bio-Rad Laboratories, Inc.). The obtained measurement data were subjected to copy-number analysis using QuantaSoft Analysis Pro software (Bio-Rad Laboratories Inc.). *RPP30* was used as the reference gene.

### Data collection from center for cancer genomics and advanced therapeutics (C-CAT) and GENIE

The C-CAT is a national datacenter for cancer genomic medicine. Clinical information and genomic data from comprehensive genomic profiling tests conducted in Japan are stored in the C-CAT [29]. The AACR Project GENIE is an international data-sharing consortium focused on generating an evidence base for precision cancer medicine by integrating clinical-grade cancer genomic data with clinical outcome data for tens of thousands of patients with cancer treated at multiple institutions worldwide [30]. The C-CAT data were accessed on Aug. 14th 2022 through the C-CAT portal. CNS/brain is the tumor type used to extract data from 498 cases of brain tumors. GENIE data were accessed on Aug. 16th 2022 through cBioPotal. In total, 8,562 glioma cases were identified. The mutation status of *IDH1/2* was used to determine GBM, IDH-wildtype.

### Statistical analysis

Information on statistical testing is provided in the description of each test in the Methods section and the corresponding result description and figure legends. No statistical method was used to pre-determine the sample size. Statistical significance was set at *p* < 0.05, except for in Fig. 2A, where statistical significance was set at *p* < 0.00625 (Bonferroni correction).

## Results

### Patient characteristics

The study cohort comprised 124 patients with gliomas who presented with a Karnofsky Performance Status (KPS) ≥ 70 and underwent surgical resection between 2005 and 2017 at the National Cancer Center in Japan. The demographic and clinical data of the patients are summarized in Table 1 and Supplementary Data 1. The median age was 39 years, which was comparable between female and male patients (mean, 59.4 vs. 62.6 years; *p* = 0.5, Student’s t-test). The diagnosis was based on the 2016 WHO classification criteria for GBM (grade IV), anaplastic astrocytomas (grade III), anaplastic oligoastrocytomas (grade III), anaplastic oligodendrogliomas (grade III), diffuse astrocytomas (grade II), oligodendrogliomas, oligoastrocytomas (grade II), pilocytic astrocytomas (grade I), and other tumors, such as anaplastic ependymomas, subependymomas, gangliogliomas, and low-grade gliomas. All patients underwent surgery, and after the initial treatment during the median follow-up time of 69.2 months, recurrence was observed in 92.7% of the patients.

**Table 1:** Demographic features of the 124 patients with glioma

	No. (%)	Age	Sex
		Median (range), years Mean (SD), years	Male no. (%) Female no. (%)
All	124	39 (5–87) 46.3 (19.4)	68 (54.8) 56 (45.2)
Diagnosis*			
Glioblastoma (grade IV) age < 40 years	15 (12.1)	31 (17–38) 30.3 (6.5)	9 (7.3) 6 (4.8)
age ≧ 40 years	38 (30.6)	67 (40–87) 65.9 (14.5)	18 (14.5) 20 (16.1)
Diffuse astrocytoma, grade II	8 (6.5)	36 (5–75) 35.6 (20)	3 (2.4) 5 (4)
Anaplastic astrocytoma, grade III	27 (21.8)	38 (17–80) 40.4 (16.2)	12 (9.7) 15 (12.1)
Oligodendroglioma, grade II	11 (8.9)	43 (31–64) 42.3 (11.7)	8 (6.5) 3 (2.4)
Oligoastrocytoma, grade II	5 (4)	31 (27–42) 33.8 (6.5)	4 (3.2) 1 (0.8)
Anaplastic oligodendroglioma, grade III	7 (5.6)	38 (28–60) 40.9 (10.7)	5 (4) 2 (1.6)
Anaplastic oligoastrocytoma, grade III	8 (6.5)	31 (28–63) 36.4 (11.8)	7 (5.6) 1 (0.8)
Pilocytic astrocytoma, grade I	2 (1.6)	30 (28–32) 30 (2.8)	1 (0.8) 1 (0.8)
Subependymoma	1 (0.8)	80 80 (ND)	0 (0) 1 (0.8)
Anaplastic ependymoma	1 (0.8)	13 13 (ND)	1 (0.8) 0 (0)
Ganglioglioma	1 (0.8)	42 42 (ND)	0 (0) 1 (0.8)

^*^The 2016 World Health Organization Classification of Tumors of the Central Nervous System

*SD* standard deviation, *ND* not determined

### Molecular profiling of gliomas by cancer gene panel sequencing

RNA-seq with TOP2-RNA was performed to detect mutations in glioma-related genes, oncogenic fusion genes, and gene overexpression (defined as > average + 3 standard deviation) for receptor tyrosine kinase (TK) (Supplementary Data 2). Initially, mutation analysis of *IDH1/2* and *H3F3A* was conducted to convert the diagnosis into one based on the 2021 WHO classification criteria. *IDH1*, *IDH2,* and *H3F3A* mutations were identified in 56, 5, and 4 cases, respectively. The molecular status was converted in the cohort into 55 cases of GBM, IDH-wildtype, grade 4; 32 cases of astrocytomas, IDH-mutant, grade 2/3/4; 27 cases of oligodendrogliomas, IDH-mutant, and 1p/19q-codeleted, grade 2/3; and 10 cases of other gliomas, including 4 cases of diffuse midline glioma, H3 K27-altered (Supplementary Fig. 1).

**Fig. 1:**
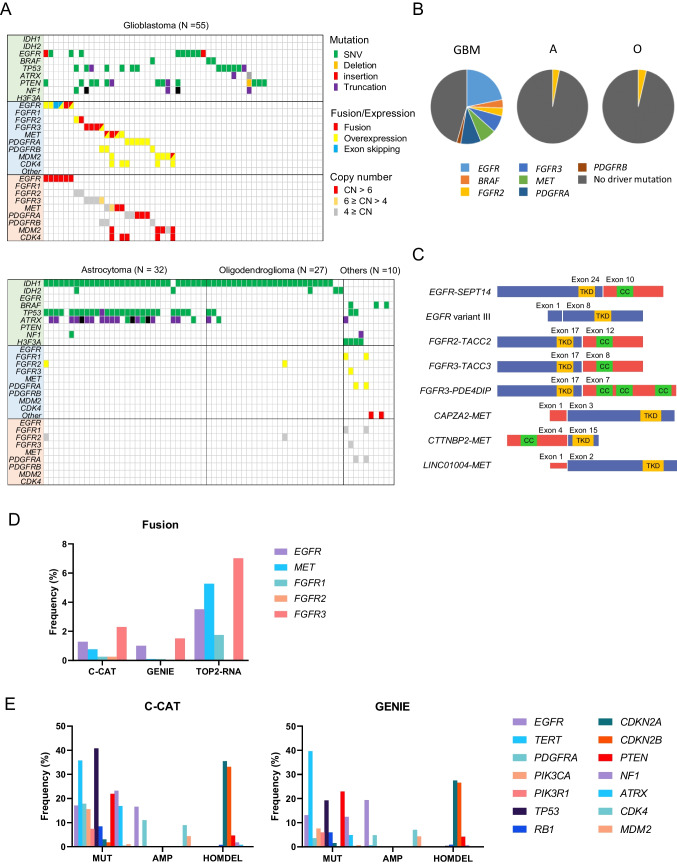
Mutational profile of gliomas. **A** Frequently mutated genes with color coding of their alteration status for each tumor. The sheet on the top (highlighted in green) shows the results of mutation analysis on *IDH1/2*, *EGFR*, *BRAF*, *TP53*, *ATRX*, *PTEN*, *NF1*, and *H3F3A*. The sheet on the middle (highlighted in blue) shows the results of fusion and exon skipping detection, and expression analysis. The sheet on the bottom (highlighted in orange) shows the results of copy-number analysis by digital droplet PCR. SNV; single nucleotide variant, CN; copy number. **B** Frequency of driver mutations in glioblastoma (GBM), astrocytomas (A), and oligodendrogliomas (O) in the cohort. **C** A schematic diagram depicting TK fusions. *EGFR*, *FGFR2, FGFR3*, and *MET* fusions identified by RNA-seq is shown with their functional domains. Exon 24 of *EGFR* (NM_005228) was ligated to exon 10 of *SEPT14* (NM_207366). *EGFR* variant III revealed the ligation of exons 1–8. Exon 17 of *FGFR2* (NM_000141) was ligated to exon 12 of *TACC2* (NM_006997). *FGFR3* (NM_000142) was disrupted downstream of exon 17 and was subsequently ligated upstream of either exon 8 of *TACC3* (NM_006342) or exon 7 of *PDE4DIP* (NM_014644). Exons 3, 15, or 2 of *MET* (NM_001324402) were ligated to exon 1 of *CAPZA2* (NM_001987), exon 4 of *CTTNBP2* (NM_033427), or exon 1 of *LINC01004* (ENST00000450686.1), respectively. The TK domain was maintained in all identified fusions. CC, coiled-coil domain; TKD, tyrosine kinase domain. **D** Frequency of fusion and exon skipping identified in the cohorts of C-CAT, GENIE, and this study (TOP2-RNA). **E** Mutation frequency of glioma-related genes in C-CAT (left) and GENIE (right). MUT, mutation; AMP, gene amplification; HOMDEL, homozygous deletion

Mutational analysis identified recurrent oncogenic mutations in GBM, including 10 cases with *EGFR* mutations and 3 with *BRAF* mutations (Fig. 1A and B). Pathogenic mutations in tumor suppressor genes such as *TP53*, *PTEN*, and *NF1* were also identified in 13, 14, and 4 GBM cases, respectively. Frequent mutations in *TP53* (*n* = 26, 81.3%) and *ATRX* (*n* = 20, 62.5%) were identified in astrocytomas (A), whereas no frequent mutations other than *IDH1/2* were identified in oligodendrogliomas. These mutation frequencies are consistent with those reported in previous studies [31].

Fusion and exon skipping analyses identified *EGFR-SEPT14*, *EGFR* variant III, *FGFR2–TACC2*, *FGFR3–PDE4DIP*, *FGFR3–TACC3*, *CAPZA2–MET*, *CTTNBP2–MET*, and *LINC01004–MET*, which retained the TK domain (Fig. 1C). *CTTNBP2-MET* and *LINC01004–MET* were novel fusion genes. *CTTNBP2–MET* contains a coiled-coil domain that promotes TK dimerization, suggesting oncogenic fusion. *LINC01004–MET* did not harbor any functional domains in *LINC01004*. The frequency of fusion genes in this study was higher than that reported in previous studies [32, 33].

Expression analysis revealed the mRNA overexpression of *EGFR*, *FGFR1*, *FGFR2*, *FGFR3*, *MET, PDGFRA*, *PDGFRB*, *MET*, *MDM2*, and *CDK4* in four, two, three, two, three, seven, three, five, and five cases, respectively (Fig. 1A and Supplementary Fig. 2). Tyrosine kinase gene overexpression was observed in 15 (27.3%) GBM cases. In contrast, gene overexpression was barely observed in astrocytomas or oligodendrogliomas. Overall, alterations in targetable driver genes were identified in 54.5% of GBM (Fig. 1B).

### Comparison of the frequency of gene fusion in GBMs in Japan and the USA

To evaluate the clinical utility of RNA-seq in GBM, the frequency of fusion genes identified in this study was compared with that of clinical sequencing cohorts in Japan and the USA. The frequency of *EGFR*, *MET*, and *FGFR1/2/3* fusions was higher in our cohort, suggesting that RNA-seq is superior to DNA-seq for fusion detection, which has been commonly used for clinical sequencing to date (Fig. 1D). The frequency of gene alterations in GBM was comparable between the GENIE and C-CAT cohorts, suggesting a comparable performance of the gene panels used in the two cohorts (Fig. 1E).

### Evaluation of the relationship between gene mutation and gene expression

**Fig. 2:**
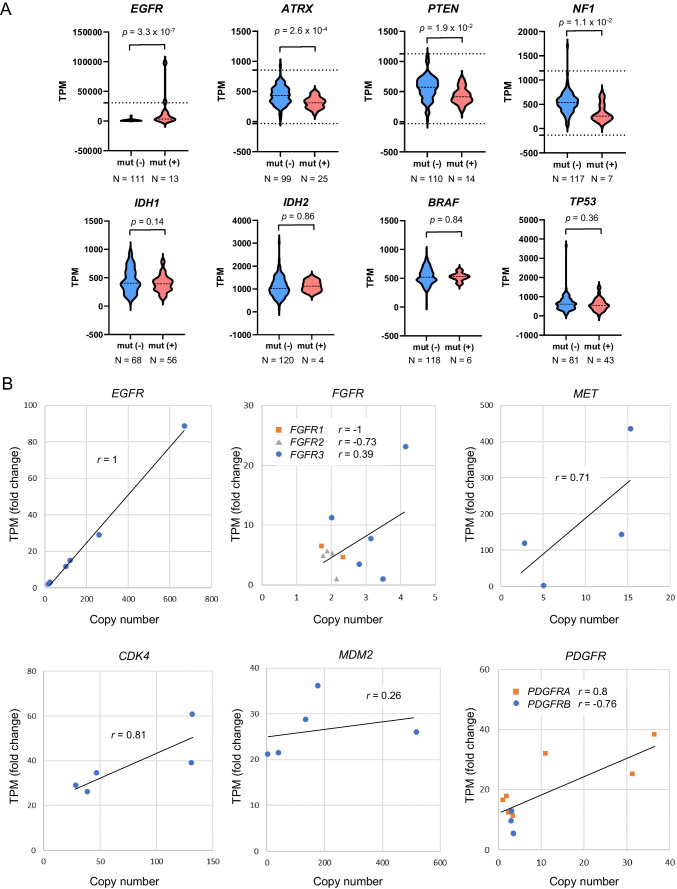
Correlation of gene expression with mutations. **A** mRNA expression was compared between cases with mutations and those without mutations in eight genes that were commonly mutated in the GBM, A and O. A high *EGFR* expression level was observed in cases with *EGFR* mutations, whereas decreased expression was observed in cases with *ATRX* mutation (*p* = 1.0 × 10^–4^, 2.0 × 10^–4^, respectively, Student’s t-test). No alteration in the expression levels of *IDH1/2*, *BRAF*, and *TP53* expression was observed between cases with and without mutations of the respective genes (*p* = 0.14, 0.86, 0.84, 0.36, respectively, Student’s t-test). The dotted line indicates threshold for outlier (> average + 3SD or < average−3SD). mut, mutation; N, number of cases. **B** Copy-number analysis by ddPCR for cases with gene overexpression. For cases with gene overexpression (defined as > average + 3SD), copy-number analysis was conducted. Gene copy number and gene expression were highly concordant in *EGFR* (*r* = 1), whereas moderate concordance was observed in *MET*, *CDK4*, and *PDGFRA* (*r* = 0.71, 0.81, and 0.79, respectively). No strong or moderate positive correlation was observed in *FGFR1/2/3*, *MDM2* and *PDGFRB* (*r* = -1, -0.73, 0.39, 0.26 and -0.76, respectively)

To evaluate the relationship between gene mutation and expression, mRNA expression was compared between cases with mutations and those without mutations in eight genes that were commonly mutated in GBM, A and O (*EGFR*, *ATRX*, *PTEN*, *NF1*, *IDH1*, *IDH2*, *BRAF*, *TP53*). A high *EGFR* expression level was observed in cases with *EGFR* mutations, whereas decreased expression was observed in cases with *ATRX* mutations (*p* = 3.3 × 10^–7^, 2.6 × 10^–4^, respectively, Student’s t-test) (Fig. 2A and Supplementary Fig. 3). No alterations in *IDH1/2*, *BRAF*, or *TP53* expression levels were observed between cases with and without mutations in the respective genes.

### Evaluation of the concordance between copy-number amplification and gene overexpression

For cases with overexpression of TK genes, *MDM2*, and *CDK4*, copy number analysis was conducted to assess if copy number amplification was the cause of gene overexpression (Supplementary Fig. 4). Gene copy number and gene expression were strongly positively correlated with *EGFR* (*r* = 1), whereas moderate positive correlations were observed for *MET*, *CDK4*, and *PDGFRA* (*r* = 0.71, 0.81, and 0.8, respectively) (Fig. 2B). No strong or moderate positive correlations were observed for *FGFR1/2/3*, *MDM2* or *PDGFRB* (*r* = -1, -0.73, 0.39, 0.26 and -0.76, respectively). Overall, among 34 cases of overexpression in GBM, 22 copy number amplifications (64.7%) were observed, suggesting the involvement of other genetic or epigenetic alterations in gene overexpression.

### Identification of prognostic biomarker of glioma by transcriptional profiling

**Fig. 3:**
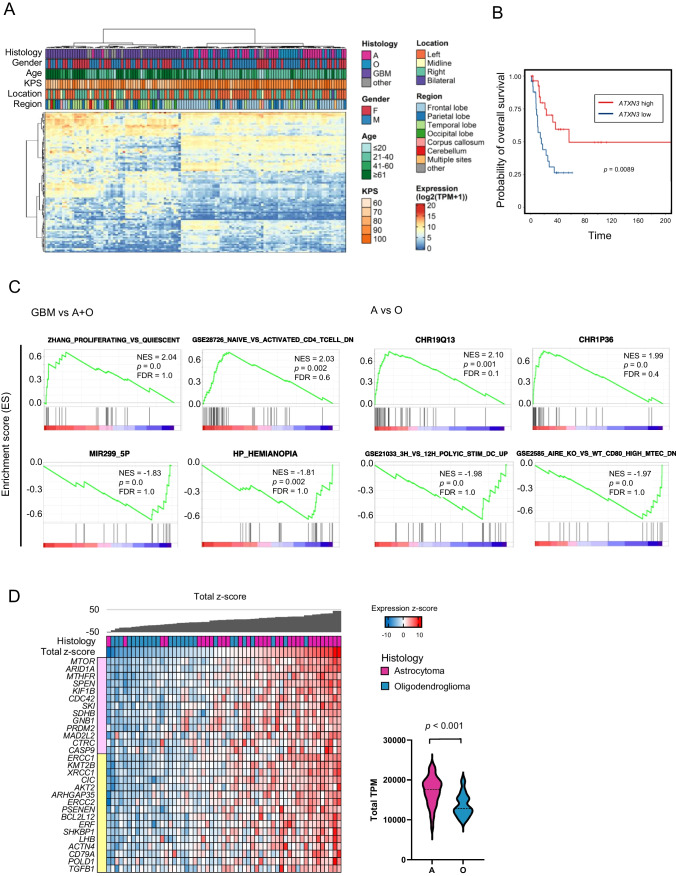
Gene expression profile of gliomas. **A** K-means clustering analysis was conducted using RNA-seq data. Clinical information (histology, gender, age, KPS, location, and region) is shown with color codes in the upper part. The RNA expression of the 100 most variable genes among samples is depicted as a heatmap at the bottom. **B** Kaplan–Meier curves of overall survival (OS) in the cohort stratified by *ATXN3* expression. Univariate Cox proportional hazards regression analysis showed *ATXN3* as the most significant correlation with OS. The survival curves were generated for the two groups, *ATXN3* high (z-score > 0) and *ATXN3* low (z-score ≤ 0), and its expression well stratified the prognosis of patients with GBM (*p* = 0.0089, log-rank test) **(C)** GSEA results with all gene sets differentially enriched among glioblastoma (GBM) vs. astrocytomas (A) + oligodendrogliomas (O) (left panel) or A vs. O (right panel) clusters defined by k-means clustering. Full GSEA results can be found in Supplementary Data 5 and 6. **D** Of 64 genes, the expression of 29 genes was moderately (0.5 < *r* ≦ 0.8) or highly (0.8 < *r*) correlated with the total z-score of gene expressions. The heat map of these 29 genes and their total z-score are shown with histology information. The right violin plot reveals that the total z-score of these 29 genes is lower in oligodendrogliomas than in astrocytomas (*p* < 0.001, Student’s t-test)

Clustering analysis was conducted using top 100 genes with the greatest variation in expression. The cohort was divided into two groups: Cluster 1 and Cluster 2 (left and right clusters in Fig. 3A). Patients with GBM were enriched in cluster 1 (*p* < 0.01, Fisher’s exact test). Most of cluster 1 was composed of GBM (46/56, 82.1%), while most of cluster 2 was composed of astrocytoma and oligodendroglioma (57/68, 83.8%), suggesting a distinct gene expression profile of GBM compared to the other subtypes of glioma. Patients with tumors located in the frontal lobe were enriched in cluster 2 (*p* < 0.01, Fisher’s exact test). Cluster 2 comprised of younger patients (*p* < 0.01 vs Cluster 1, Student’s t-test). The median overall survival (OS) period was 56.7 and 87.4 months for clusters 1 and 2, respectively, and was not significantly different (*p* = 0.38, log-rank test; 95% confidence interval [CI] of ratio = 0.37–1.14 and 0.87–2.71) (Supplementary Fig. 5A).

The data are bulk transcriptomes, and the two clusters reflect the microenvironment composition of the individual tumors. The expression of macrophage marker genes *CD68* and *CD163* or genes related to T-cell activation, namely *CCL5*, *CD58*, and *CXCL10,* was elevated specifically in cluster 1, suggesting immune cell activation of tumors in cluster 1 (Supplementary Fig. 5B and Supplementary Data 3).

Risk of 55 GBM patients was assessed using RNA-seq data. The median OS period was 32.8 (95% CI = 21.3–NA) months. Univariate Cox proportional hazards regression analysis showed a significant correlation between the seven genes and OS (*p* ≤ 1 × 10^–3^, log-rank test) (Supplementary Data 4). The most significant gene was *ATXN3*, and its expression strongly stratified the prognosis of patients with GBM (*p* = 0.0089, log-rank test; 95% confidence interval [CI] ratio = 32.8–NA and 10.1–NA) (Fig. 3B).

### Expression of genes on chr1p36 and chr19q13 is a diagnostic marker to distinguish oligodendroglioma

Gene set enrichment analysis (GSEA) between GBMs and astrocytomas or oligodendrogliomas identified 26 and 25 gene sets enriched in each group (Nominal *p* value < 0.01, one cluster vs. the other) (Supplementary Data 5). Among these gene sets, “ZHANG_PROLIFERATING_VS_QUIESCENT” and “GSE28726_NAIVE_VS_ACTIVATED_CD4_TCELL_DN” were enriched in GBM, whereas “MIR299_5P” and “HP_HEMIANOPIA” were enriched in astrocytomas and oligodendrogliomas (Fig. 3C). GSEA between astrocytomas and oligodendrogliomas identified 11 and 19 upregulated gene sets, respectively (*p* < 0.01, one cluster vs. the others) (Supplementary Data 6). Among these gene sets, “CHR19Q13” and “CHR1P36” were enriched in astrocytomas, whereas “GSE21033_3H_VS_12H_POLYIC_STIM_DC_UP” and “GSE2585_AIRE_KO_VS_WT_CD80_HIGH_MTEC_DN” were enriched in oligodendrogliomas (Supplementary Fig. 6).

Of the 1,390 genes targeted by TOP2-RNA, 28 and 36 were located at the chr1p36 and chr19q13 loci, respectively. The correlation between the z-scores of individual genes and the total z-score among the samples was calculated. Of the 64 genes, the expression levels of 29 genes were moderately (0.5 < *r* ≤ 0.8) or highly (0.8 < *r*) correlated with total gene expression. The total z-score and average TPM for the 29 genes of oligodendrogliomas were lower than those of astrocytomas, indicating that the co-deletion of chr1p36 and chr19q13 led to decreased expression of genes located at the locus (*p* < 0.001, Student’s t-test) (Fig. 3D and Supplementary Fig. 7), indicating that the z-score of these genes may be applicable as a diagnostic marker for oligodendroglioma.

## Discussion

This is the first study to evaluate the feasibility and utility of RNA-seq for the detection of fusion genes and aberrant transcription in malignant gliomas using TOP2-RNA. The utility of RNA-seq has been demonstrated in the detection of fusion genes. Specifically, we identified targetable *FGFR2/3* and *MET* fusions in nine cases (16.4% of GBM). In terms of biological sampling, including ethnicity, our cohort is the same as the C-CAT cohort, suggesting that the difference in the frequency of fusion is related to the technologies used. The method used in the C-CAT and GENIE cohorts is DNA-seq, whereas our method uses RNA-seq, which is more suitable for fusion detection [34]. Additionally, *KIAA1549*-*BRAF* and *C11orf95*-*RELA* were found in cases of pilocytic astrocytoma and subependymoma, respectively (data not shown). There have been recent developments and approvals of therapies targeting fusion oncogenes, such as those for cholangiocarcinomas with *FGFR2* fusions and solid tumors with *NTRK* fusions in the area.

*CAPZA2–MET* and *LINC01004–MET* fusions did not have dimerization domains, which are usually found in TK fusion genes and promote constitutive kinase activation. However, *MET* was overexpressed in both the samples. Copy-number amplification (copy number = 15.3) was identified in samples with *LINC01004–MET,* suggesting the involvement of gene rearrangement in amplification. A recent study reported that *LINC01004* is a novel super-enhancer-associated lncRNA and crucial oncogene in hepatocellular carcinoma [35]. Therefore, promoter swapping by *LINC01004* may further promote *MET* overexpression and augment the transformation potential of this fusion.

In contrast, copy number was normal in the sample with *CAPZA2-MET*, suggesting that *MET* overexpression was caused via promoter swapping by CAPZA2. A recent study reported a patient with cholangiocarcinoma harboring a *CAPZA2–MET* fusion along with *MET* amplification, who dramatically responded to capmatinib, a specific MET TK inhibitor [36].

In addition, *EGFR* VIII was detected using an analytical pipeline to detect exon skipping. Considering that the identification of gene alterations affecting relatively long regions of the genome (> 100 nt) by short-read sequencing is difficult, the detection of exon skipping caused by structural variations is another advantage of RNA-seq.

This study evaluated the utility of expression analysis using RNA-seq and successfully identified *TK* overexpression cases. Subsequent ddPCR analysis showed that approximately half of the cases with gene overexpression harbored gene amplification. Specifically, *EGFR* RNA expression and copy number amplification were highly concordant, suggesting that genetic control is dominant for *EGFR* expression in gliomas.

The expression analysis distinguished GBMs from astrocytomas or oligodendrogliomas, suggesting distinctive features of the microenvironment composition of GBM. Across cohorts, transcriptome analyses of human GBM have repeatedly been classified into three subtypes: classic (CL), mesenchymal (MES), and proneural (PN) [37–40]. Several studies have established correlations between subtype-specific gene expression signatures, differential response to therapy, and overall patient survival; the latter is poor in highly mesenchymal tumors that exhibit innate immune cell infiltration at recurrence [40]. In this study, the comparison of RNA expression with prognosis identified several genes, namely, *ATXN3*, *GOLGA5*, *CRBN*, *MAX*, *TGFB3*, *SETD3,* and *SFTPA1,* as prognostic markers that may be related to the mesenchymal subtype.

*ATXN3* (Ataxin 3) encodes a deubiquitinating enzyme involved in protein homeostasis, transcription, cytoskeleton regulation, myogenesis, and degradation of misfolded chaperone substrates [41–44]. *ATXN3-*associated diseases occur as Machado–Joseph disease and Machado–Joseph disease type 1, which is an autosomal dominant neurodegenerative disorder predominantly involving the cerebellar, pyramidal, extrapyramidal, motor neuron, and oculomotor systems. A recent study reported that *ATXN3* is targeted by piRNAs and miRNAs and that its upregulation might induce cell proliferation through G-protein-coupled receptor or AKT signaling in GBM [45].

Although the methylation status of the *MGMT* promoter was positively correlated with prolonged survival in patients who received TMZ-based therapy [8], *MGMT* mRNA expression was not significantly related to OS in our cohort (*p* = 0.32, hazard ratio = 1.24). Further studies on the correlation between *MGMT* promoter status and the prognosis of IDH-wildtype GBM are needed.

Another utility of TOP2-RNA is its diagnostic capability in astrocytomas and oligodendrogliomas. Although oligodendrogliomas are associated with *CIC* mutations (up to 70%) [46, 47], astrocytomas frequently harbor *TP53* and *ATRX* mutations [48] and confirmation of 1p/19q co-deletion is needed to distinguish both tumors according to the 2021 WHO classification. The assessment of gene expression on the 1p/19q locus with TOP2-RNA may be used as a substitute for fluorescence in situ hybridization or SNP arrays, which are common assays in routine clinical settings. Although copy-number variants such as 1p/19q co-deletion by whole genome sequencing, exome sequencing, and targeted NGS assays have been explored in brain tumors using various bioinformatics analysis pipelines [49–54], few studies have reported the utility of targeted RNA-seq for 1p/19q co-deletion.

In addition to 1p/19q co-deletion in oligodendrogliomas, GSEA revealed intriguing features of glioblastoma. For instance, ZHANG_PROLIFERATING_VS_QUIESCENT is upregulated in proliferating HDMEC cells (microvascular endothelium). This is a reasonable result considering that GBM is characterized by extensive vascularization, and its tumor angiogenesis is known to be a multi-step process involving the proliferation, migration, and differentiation of brain microvascular endothelial cells under the stimulation of specific signals derived from cancer cells [55]. GSE28726_NAIVE_VS_ACTIVATED_CD4_TCELL_DN was downregulated in activated CD4 + T cells. This result suggests that GBM has a specific immunosuppressive microenvironment as it has a distinct pattern of genomic aberrations that could be neoantigens. A recent study reported that T cell dysfunction in the glioblastoma microenvironment is mediated by myeloid cells [56]. Combining single-cell RNA sequencing of the immune compartment with spatially resolved transcriptomic sequencing in different types of glioma will deepen our understanding of how GBM creates an immunosuppressive microenvironment.

This study had several limitations. First, DNA analysis was not performed to comprehensively assess mutational profiles. Although mutational analysis by RNA-seq may overlook mutations in low-expression genes, hotspot mutations in oncogenes such as *IDH1/2* were successfully identified, and cases with *IDH1/2* mutations were highly consistent with the pathological diagnosis. Second, the cause of the gene overexpression was not fully identified. Approximately half of the cases with high gene expression showed copy number amplification. The structural rearrangements that cause promoter or enhancer swapping may be involved in the other half. Thus, whole-genome sequencing may help elucidate the underlying causes of gene overexpression. Third, this study only evaluated fresh-frozen specimens, which is not common in the pathology department. In our previous study, we validated the capability of TOP-RNA for fusion detection using 38 FFPE specimens of non-small cell lung cancer and sarcoma, which were confirmed to harbor fusion genes in fresh-frozen samples. TOP-RNA detected the respective fusions in all 38 samples, including small biopsy specimens [27]. Expression analysis was also conducted for seven tumors to compare the performance of the TOP RNA panel using FFPE specimens with that of poly(A)-RNA-seq using frozen specimens. The mRNA expression values of the 109 genes in the TOP RNA Panel were highly concordant with those determined by poly(A)-RNA sequencing, even though the former data were obtained using FFPE specimens (*r* = 0.94–0.99). Fourth, we did not have information on the copy numbers of chr1p and 19q. A confirmatory study to evaluate the concordance between FISH and TOP-RNA remains to be conducted.

This study confirms that TOP2-RNA is a highly sensitive assay for detecting fusion genes, exon skipping, and aberrant gene expression. We identified alterations in targetable driver genes in more than 50% of GBM cases. Above, expression profiling has identified several candidate markers that could directly predict the prognosis of GBM. Expression analysis also suggested that TOP2-RNA could precisely differentiate oligodendroglioma from astrocytoma. In summary, molecular profiling by TOP2-RNA provides ample predictive, prognostic, and diagnostic biomarkers that may not be identified by conventional assays, and therefore, increases treatment options for patients with gliomas.

### Supplementary Information

Below is the link to the electronic supplementary material.

Supplementary file1 (XLSX 2130 KB)

ESM 1 Supplementary Figure 1. Diagnosis conversion into the WHO 2021 classification criteria. Mutation analysis of *IDH1/2* and *H3F3A* was conducted based on the WHO 2021 classification criteria to convert the diagnosis into one. The molecular status converted the cohort into 55 cases of GBM, IDH-wildtype, grade 4; 32 cases of astrocytomas, IDH-mutant, grade 2/3/4; 27 cases of oligodendrogliomas, IDH-mutant and 1p/19q-codeleted, grade 2/3; and 10 cases of other gliomas, including four cases of diffuse midline glioma, H3 K27-altered. Supplementary Figure 2. Expression analysis of glioma-related oncogenes. Expression analysis identified the mRNA overexpression of *EGFR, FGFR1, FGFR2, FGFR3, MET, PDGFRA, PDGFRB, MET, MDM2*, and* CDK4* in four, two, three, two, three, seven, three, five, and five cases, respectively. Samples with gene overexpression (defined as >average + 3SD) are circled in red. Supplementary Figure 3. mRNA expression of *EGFR, ATRX, PTEN* and *NF1* without outlier cases. mRNA expression was compared between patients with and without mutations in *EGFR, ATRX, PTEN* and *NF1*. Cases with outlier expression (>average + 3SD or <average - 3SD) were excluded. A high *EGFR* expression level was observed in cases with *EGFR* mutations, whereas decreased expression was observed in cases with *ATRX* mutations (p = 1.0 × 10^-11^, 2.4 × 10^-4^, respectively, Student’s t-test). The dotted line indicates the threshold for outliers (>average + 3SD or <average - 3SD). Supplementary Figure 4. Copy-number analysis using ddPCR. The copy numbers (CNs) of *CDK4, EGFR, FGFR1, FGFR2, FGFR3, MDM2, ERBB2, MET, PDGFRA*, and *PDGFRB* obtained using ddPCR are shown in a two-dimensional plot. The X-axis indicates the amplitude of the target genes (Ch1), whereas the y-axis indicates the amplitude of the reference gene (Ch2). The black clusters in the plots represent both negative droplets (Ch1− and Ch2−), green clusters represent droplets positive for the *RPP30* reference gene (Ch1− and Ch2+), blue clusters represent droplets positive for target genes (Ch1+ and Ch2−), and orange clusters represent droplets positive for both genes (Ch1+ and Ch2+). CNs were calculated based on the number of copies of the target genes, assuming that the reference gene had two copies. Supplementary Figure 5. Comparison of clusters 1 and 2 in prognosis and gene expression. (**A**) Kaplan–Meier curves of overall survival in the cohort stratified by k-means clustering as clusters 1–2. (**B**) GSEA results with the indicated gene sets differentially enriched among clusters defined by k-means clustering. The full GSEA results can be found in Supplementary Data 3. Supplementary Figure 6. Heatmap of the expression of the genes on chr19q13 and chr1p36. The heatmap revealed the expression of 36 genes on chr19q13 and 28 genes on chr1p36 across astrocytomas and oligodendrogliomas. Supplementary Figure 7. Heatmap of the TPM for 29 genes on chr19q13 and chr1p36. The heat map shows the TPM for 29 genes on chr19q13 and chr1p36 and their average TPM with histological information. The violin plot revealed that the average TPM of these 29 genes was lower in oligodendrogliomas than that in astrocytomas (*p* = 0.0001, Student’s t-test). (PPTX 2474 KB)

## Data Availability

We have deposited the raw sequencing data in the Japanese Genotype-Phenotype Archive, which is hosted by the DNA Data Bank of Japan, under accession number JSUB000932.

## Abbreviations

TOP2-RNA: Todai OncoPanel 2 RNA Panel
RNA-seq: RNA-sequencing
GSEA: Gene set enrichment analysis
ddPCR: Digital droplet polymerase chain reaction
IDH: Isocitrate dehydrogenase
GBM: Glioblastomas
WHO: World Health Organization
TPM: Transcripts per million
KPS: Karnofsky Performance Status
TK: Tyrosine kinase
A: Astrocytomas
OS: Overall survival
CI: Confidence interval
