# Supplementary material for: The development of a custom RNA-sequencing panel for the identification of predictive and diagnostic biomarkers in glioma
Source: J Neurooncol. 2024 Feb 16;167(1):75–88. doi: 10.1007/s11060-024-04563-z (PMC10978676; doi:10.1007/s11060-024-04563-z)
Supplement: Supplementary file 2 — ESM 1 Supplementary Figure 1. Diagnosis conversion into the WHO 2021 classification criteria. Mutation analysis of IDH1/2 and H3F3A was conducted based on the WHO 2021 classification criteria to convert the diagnosis into one. The molecular status converted the cohort into 55 cases of GBM, IDH-wildtype, grade 4; 32 cases of astrocytomas, IDH-mutant, grade 2/3/4; 27 cases of oligodendrogliomas, IDH-mutant and 1p/19q-codeleted, grade 2/3; and 10 cases of other gliomas, including four cases of diffuse midline glioma, H3 K27-altered. Supplementary Figure 2. Expression analysis of glioma-related oncogenes. Expression analysis identified the mRNA overexpression of EGFR, FGFR1, FGFR2, FGFR3, MET, PDGFRA, PDGFRB, MET, MDM2, and CDK4 in four, two, three, two, three, seven, three, five, and five cases, respectively. Samples with gene overexpression (defined as >average + 3SD) are circled in red. Supplementary Figure 3. mRNA expression of EGFR, ATRX, PTEN and NF1 without outlier cases. mRNA expression was compared between patients with and without mutations in EGFR, ATRX, PTEN and NF1. Cases with outlier expression (>average + 3SD or <average - 3SD) were excluded. A high EGFR expression level was observed in cases with EGFR mutations, whereas decreased expression was observed in cases with ATRX mutations (p = 1.0 × 10^-11^, 2.4 × 10^-4^, respectively, Student’s t-test). The dotted line indicates the threshold for outliers (>average + 3SD or <average - 3SD). Supplementary Figure 4. Copy-number analysis using ddPCR. The copy numbers (CNs) of CDK4, EGFR, FGFR1, FGFR2, FGFR3, MDM2, ERBB2, MET, PDGFRA, and PDGFRB obtained using ddPCR are shown in a two-dimensional plot. The X-axis indicates the amplitude of the target genes (Ch1), whereas the y-axis indicates the amplitude of the reference gene (Ch2). The black clusters in the plots represent both negative droplets (Ch1− and Ch2−), green clusters represent droplets positive for the RPP30 reference gene (Ch1− and Ch2+), blue clusters re [file 11060_2024_4563_MOESM2_ESM.pptx]

## Slide 1
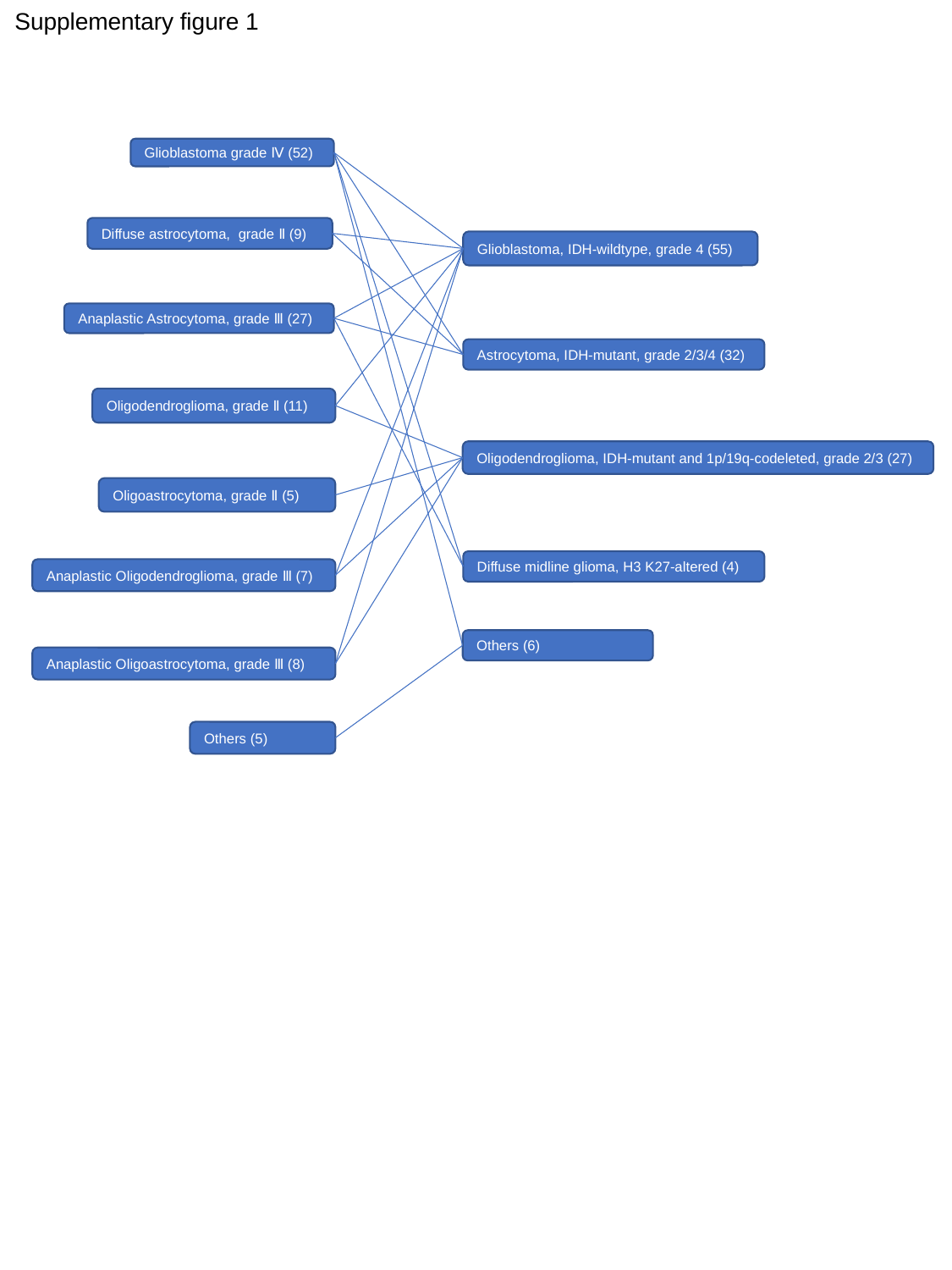

Supplementary figure 1
Glioblastoma grade Ⅳ (52)
Diffuse astrocytoma, grade Ⅱ (9)
Glioblastoma, IDH-wildtype, grade 4 (55)
Anaplastic Astrocytoma, grade Ⅲ (27)
Astrocytoma, IDH-mutant, grade 2/3/4 (32)
Oligodendroglioma, grade Ⅱ (11)
Oligodendroglioma, IDH-mutant and 1p/19q-codeleted, grade 2/3 (27)
Oligoastrocytoma, grade Ⅱ (5)
Diffuse midline glioma, H3 K27-altered (4)
Anaplastic Oligodendroglioma, grade Ⅲ (7)
Others (6)
Anaplastic Oligoastrocytoma, grade Ⅲ (8)
Others (5)

## Slide 2
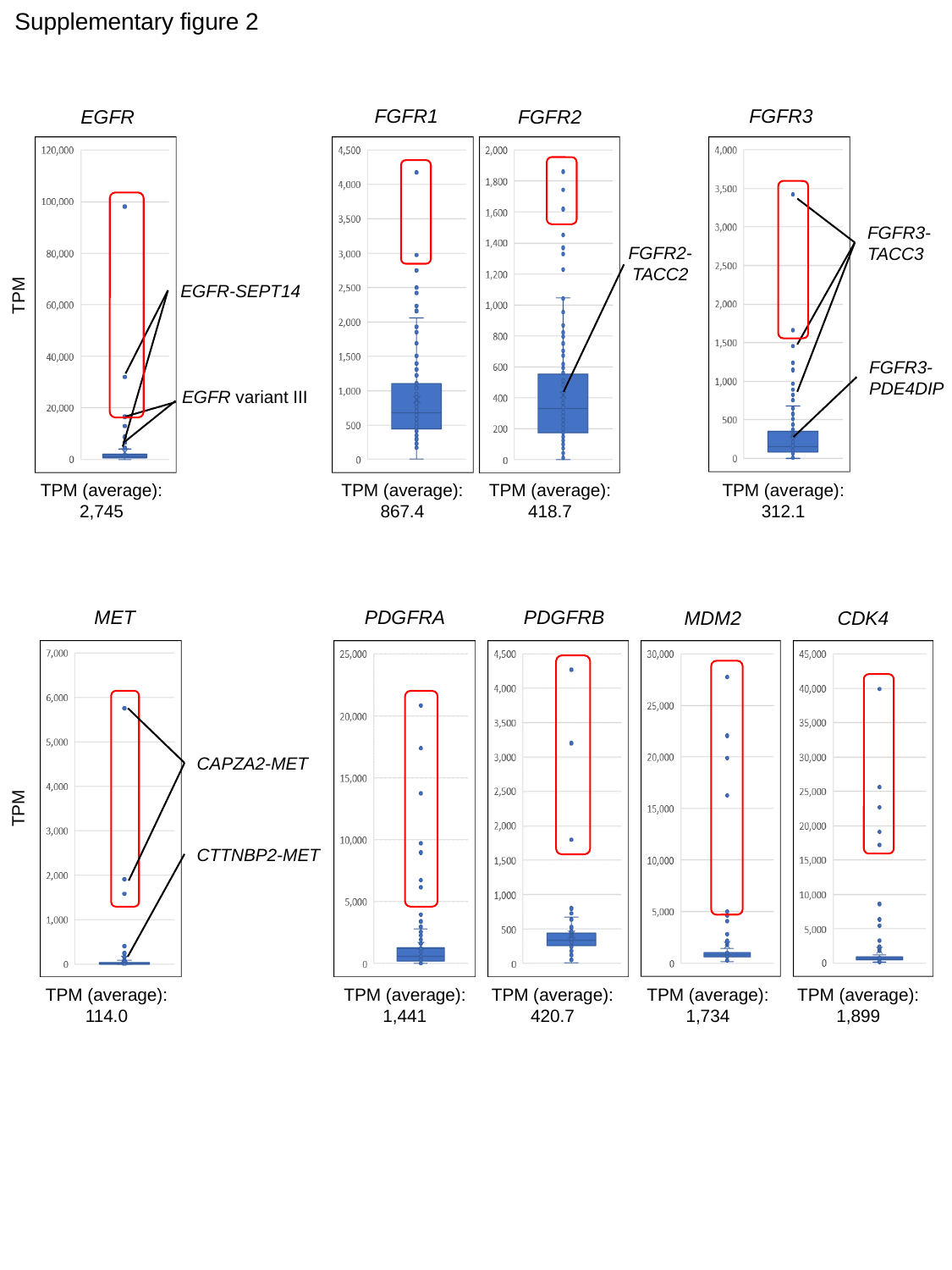

Supplementary figure 2
FGFR1
FGFR3
FGFR2
 EGFR
FGFR3-TACC3
FGFR2-TACC2
EGFR-SEPT14
TPM
FGFR3-PDE4DIP
EGFR variant III
TPM (average): 2,745
TPM (average): 867.4
TPM (average): 418.7
TPM (average): 312.1
MET
PDGFRA
PDGFRB
MDM2
CDK4
CAPZA2-MET
TPM
CTTNBP2-MET
TPM (average): 114.0
TPM (average): 1,441
TPM (average): 420.7
TPM (average): 1,734
TPM (average): 1,899

## Slide 3
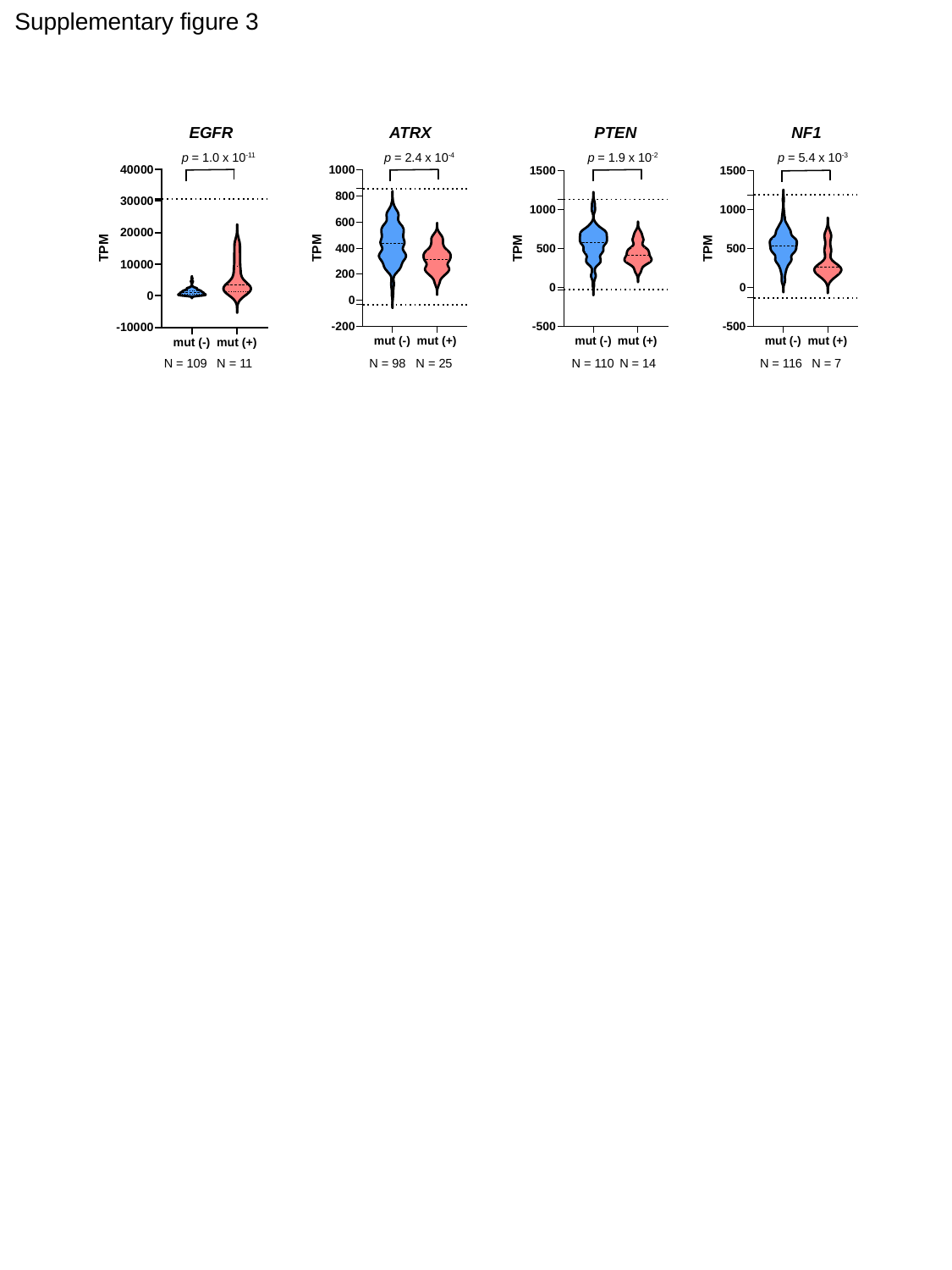

Supplementary figure 3
EGFR
ATRX
PTEN
NF1
p = 1.0 x 10^-11^
p = 2.4 x 10^-4^
p = 1.9 x 10^-2^
p = 5.4 x 10^-3^
N = 109
N = 11
N = 98
N = 25
N = 110
N = 14
N = 116
N = 7

## Slide 4
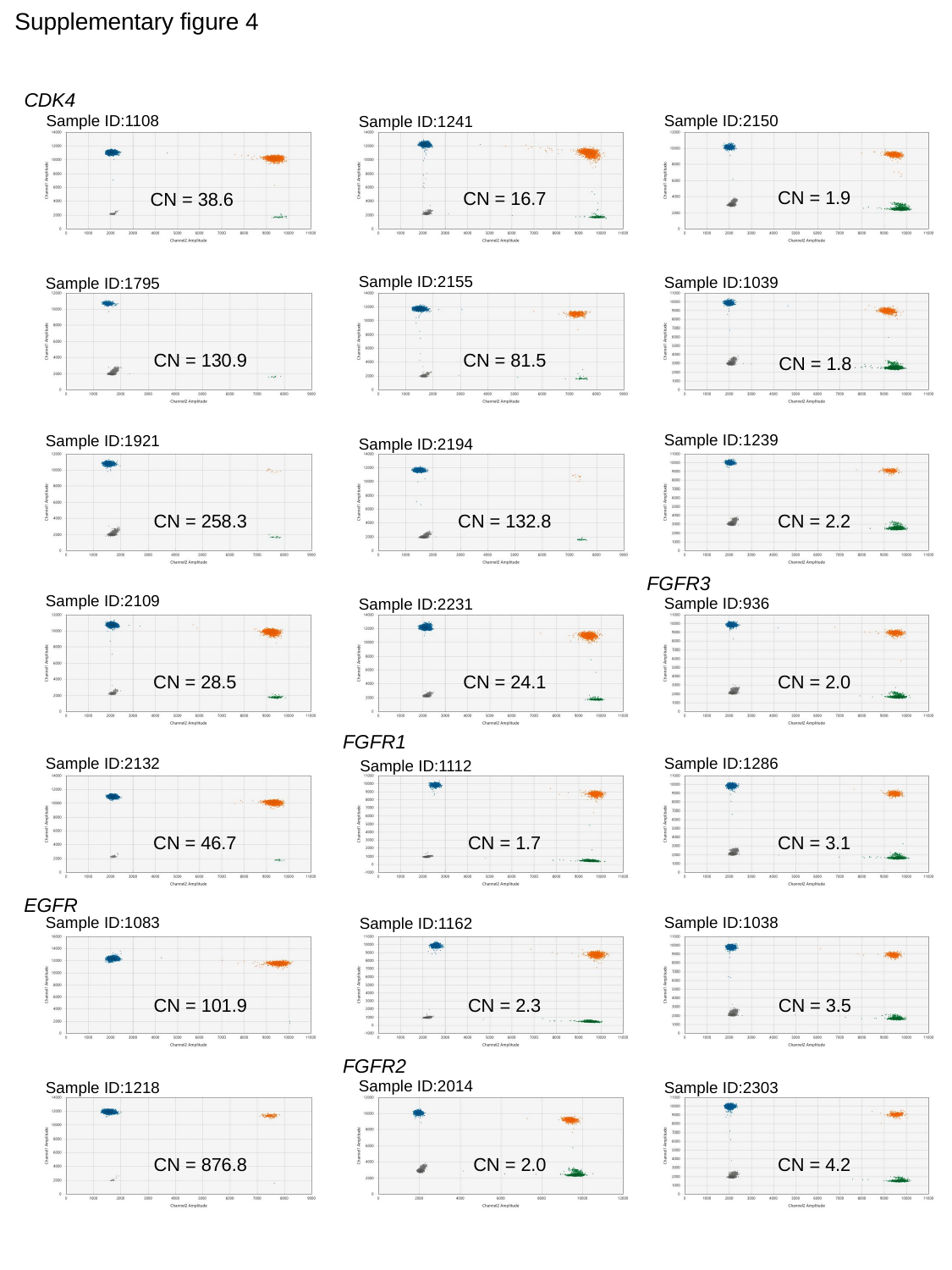

Supplementary figure 4
CDK4
Sample ID:1108
Sample ID:2150
Sample ID:1241
CN = 1.9
CN = 16.7
CN = 38.6
Sample ID:2155
Sample ID:1039
Sample ID:1795
CN = 81.5
CN = 130.9
CN = 1.8
Sample ID:1239
Sample ID:1921
Sample ID:2194
CN = 258.3
CN = 132.8
CN = 2.2
FGFR3
Sample ID:2109
Sample ID:936
Sample ID:2231
CN = 28.5
CN = 24.1
CN = 2.0
FGFR1
Sample ID:2132
Sample ID:1286
Sample ID:1112
CN = 46.7
CN = 1.7
CN = 3.1
EGFR
Sample ID:1038
Sample ID:1083
Sample ID:1162
CN = 101.9
CN = 2.3
CN = 3.5
FGFR2
Sample ID:2014
Sample ID:1218
Sample ID:2303
CN = 876.8
CN = 2.0
CN = 4.2

## Slide 5
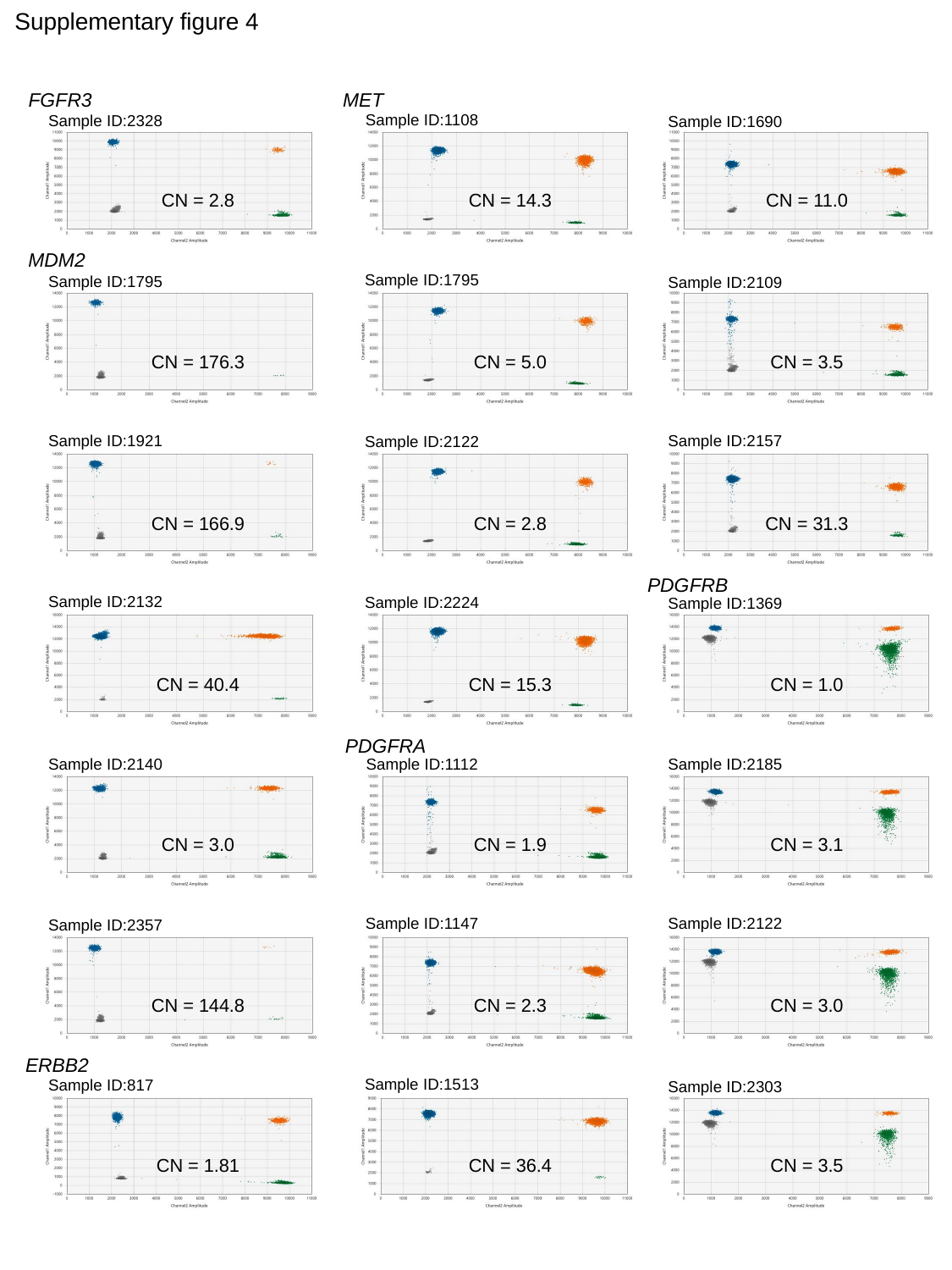

Supplementary figure 4
FGFR3
MET
Sample ID:1108
Sample ID:2328
Sample ID:1690
CN = 2.8
CN = 14.3
CN = 11.0
MDM2
Sample ID:1795
Sample ID:1795
Sample ID:2109
CN = 176.3
CN = 5.0
CN = 3.5
Sample ID:2157
Sample ID:1921
Sample ID:2122
CN = 166.9
CN = 2.8
CN = 31.3
PDGFRB
Sample ID:2132
Sample ID:2224
Sample ID:1369
CN = 40.4
CN = 15.3
CN = 1.0
PDGFRA
Sample ID:2140
Sample ID:2185
Sample ID:1112
CN = 3.0
CN = 1.9
CN = 3.1
Sample ID:1147
Sample ID:2122
Sample ID:2357
CN = 144.8
CN = 2.3
CN = 3.0
ERBB2
Sample ID:1513
Sample ID:817
Sample ID:2303
CN = 1.81
CN = 36.4
CN = 3.5

## Slide 6
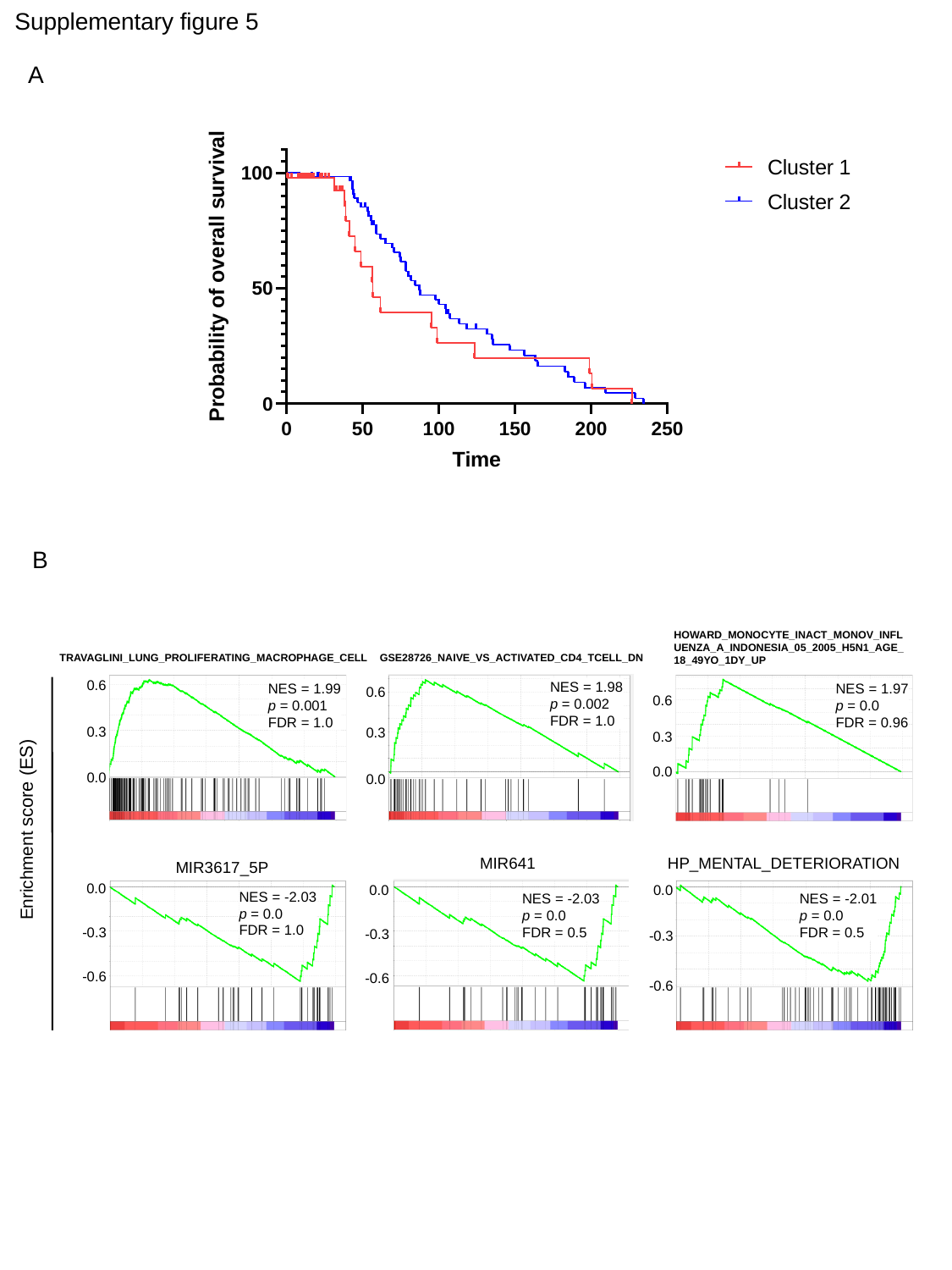

Supplementary figure 5
A
B
HOWARD_MONOCYTE_INACT_MONOV_INFLUENZA_A_INDONESIA_05_2005_H5N1_AGE_18_49YO_1DY_UP
| TRAVAGLINI\_LUNG\_PROLIFERATING\_MACROPHAGE\_CELL |
| --- |
| GSE28726\_NAIVE\_VS\_ACTIVATED\_CD4\_TCELL\_DN |
| --- |
0.6
0.3
0.0
0.6
0.3
0.0
NES = 1.98
p = 0.002
FDR = 1.0
NES = 1.99
p = 0.001
FDR = 1.0
NES = 1.97
p = 0.0
FDR = 0.96
0.6
0.3
0.0
Enrichment score (ES)
| MIR641 |
| --- |
| HP\_MENTAL\_DETERIORATION |
| --- |
| MIR3617\_5P |
| --- |
0.0
-0.3
-0.6
0.0
-0.3
-0.6
0.0
-0.3
-0.6
NES = -2.03
p = 0.0
FDR = 1.0
NES = -2.01
p = 0.0
FDR = 0.5
NES = -2.03
p = 0.0
FDR = 0.5

## Slide 7
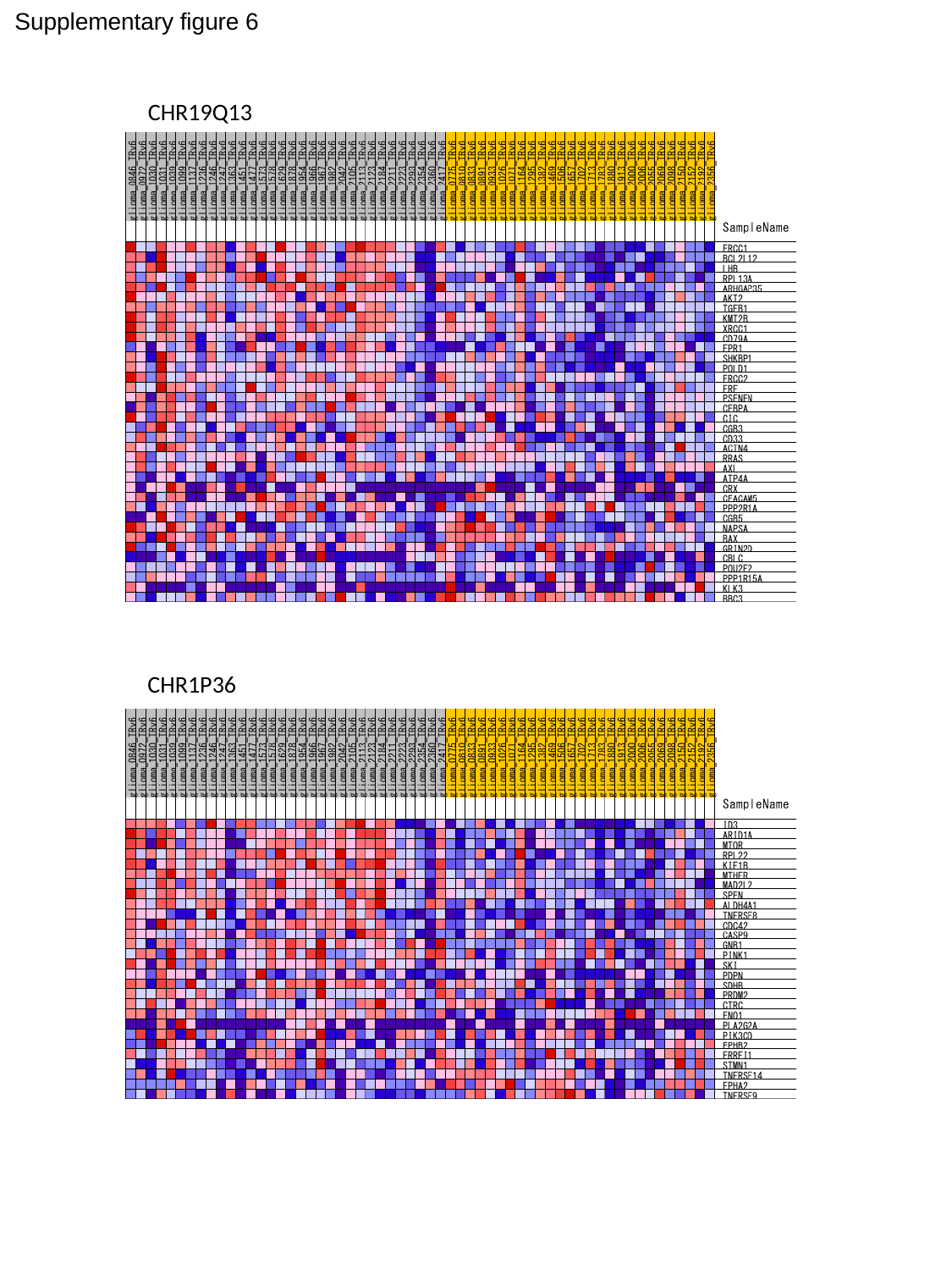

Supplementary figure 6
CHR19Q13
CHR1P36

## Slide 8
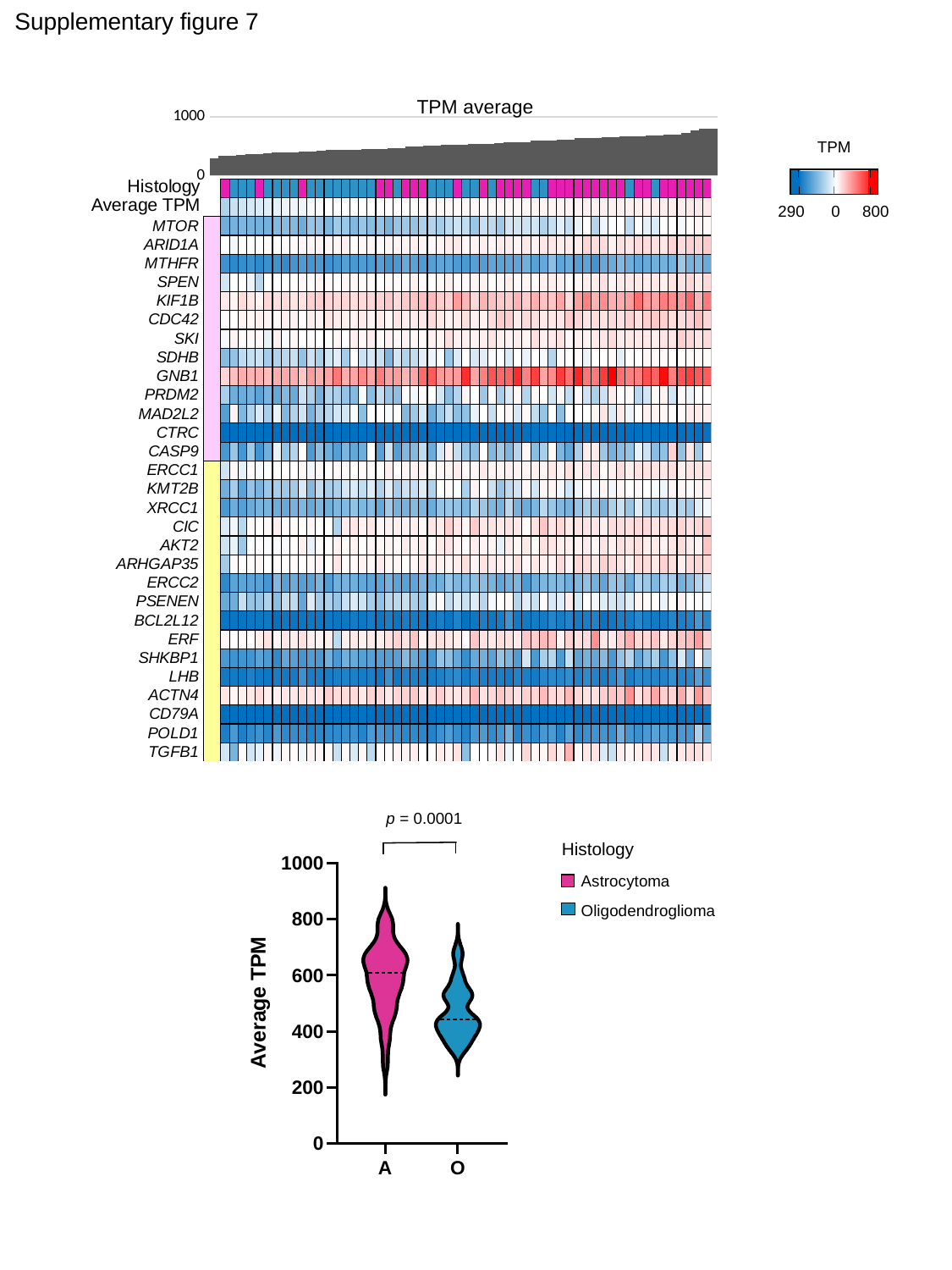

Supplementary figure 7
TPM average
### Chart
| Category | |
|---|---|TPM
290 0 800
p = 0.0001
Histology
Astrocytoma
Oligodendroglioma
